# Dunglison's Dictionary of Medical Science. Revised Edition

**Published:** 1874-04

**Authors:** 


### BIBLIOGRAPHICAL.

---

*A Dictionary of Medical Science*, containing a concise explanation of the various subjects and terms of Anatomy, Physiology, Pathology, Hygiene, Therapeutics, Medical Chemistry, Pharmacology, Pharmacy, Surgery, Obstetrics, Medical Jurisprudence, and Dentistry. Notices of climate and mineral waters, formulа for officinal, empirical and dietetic preparations ; with the accentuation and etymology of the terms, and the French and othor synonyms. By Robley Dunglison, M. D., L. L. D. A new edition enlarged and thoroughly revised by Richard J. Dunglison, M. D. Published by Henry C. Lea, Philadelphia, 1874.

The new edition of this old and reliable text book will meet with a cordial welcome, as no less than six thousand terms and subjects have been added, and the type so enlarged as to greatly enhance the value of the work, and bring it up to the requirements of the day. The typographical arrangement of this new edition renders reference much more easy, and the care devoted to the accentuation as well as derivation of terms makes it a complete pronouncing dictionary.

